# Effect of pictorial-based information about atherosclerosis on adherence to lifestyle recommendations: results from the VIPVIZA randomised controlled trial

**DOI:** 10.1136/openhrt-2026-004136

**Published:** 2026-07-23

**Authors:** Andree Christian Wennstig, Sara Själander, Andreas Hult, Per Liv, Margareta Norberg, Maria Wennberg, Per Wester, Patrik Wennberg

**Affiliations:** 1 Department of Public Health and Clinical Medicine, Umeå University, Umeå, Sweden; 2 Department of Community Medicine and Rehabilitation, Umeå University, Umeå, Sweden

**Keywords:** Risk Factors, Atherosclerosis, Carotid Arteries, Health Services, Ultrasonography

## Abstract

**Aim:**

Low adherence to a healthy lifestyle constitutes a major barrier to effective prevention of cardiovascular disease. We aimed to investigate the effect of pictorial-based information about subclinical atherosclerosis on adherence to health-promoting lifestyle recommendations.

**Methods:**

Visualization of Asymptomatic Atherosclerotic Disease for Optimum Cardiovascular Prevention (VIPVIZA) is a pragmatic, open-label randomised controlled trial within the Västerbotten Intervention Programme (VIP) in Sweden. In the VIP, residents of Västerbotten county are invited to a health survey and a health-promoting dialogue at the ages of 40, 50 and 60.

A total of 3532 VIP participants were included in VIPVIZA during 2013–2016. They underwent a carotid ultrasound examination in addition to the usual VIP process and were randomised 1:1 to intervention group (receiving graphical colour-coded and age-related information about carotid intima-media thickness and plaques, written information about atherosclerosis and a motivational dialogue) or control group (no information or dialogue).

We developed a lifestyle index based on self-reported physical activity, smoking habits, diet and alcohol consumption, ranging from 4 to 12, with higher levels indicating healthier lifestyles. We used ordinal regression to compare the lifestyle index in the intervention and control groups at the 3-year follow-up, with adjustments for baseline levels of lifestyle index, age, sex and education.

**Results:**

The OR for having a healthier lifestyle index after 3 years was 1.20 (95% CI 1.01 to 1.42, p=0.03), comparing the intervention group to the control group. We found no interactions for age, sex or education.

**Conclusion:**

Our findings provide some evidence for the contributory role of pictorial-based information about subclinical atherosclerosis in promoting sustained healthy lifestyle habits.

**Trial registration number:**

NCT01849575.

**WHAT IS ALREADY KNOWN ON THIS TOPIC**

- Low and non-sustained adherence to lifestyle recommendations constitutes a major barrier to effective prevention of cardiovascular disease (CVD). Existing studies of interventions in which individuals are provided with pictorial information about their CVD risk, in conjunction with efforts to motivate lifestyle change, have reported mixed or uncertain long-term results.

**WHAT THIS STUDY ADDS**

- This study demonstrates that an intervention based on pictorial information about individuals’ subclinical atherosclerosis, delivered in addition to a CVD prevention programme in primary care, improved adherence to lifestyle recommendations, measured by a lifestyle index, after 3 years.

**HOW THIS STUDY MIGHT AFFECT RESEARCH, PRACTICE OR POLICY**

- Our findings indicate that personalised ultrasound-based visualisation of subclinical atherosclerosis delivered in primary care and supported by behavioural change techniques can enhance long-term adherence to healthy lifestyle behaviours. This approach represents a promising direction for future research, including implementation studies examining how the methodology can be integrated into preventive care programmes.

## Introduction

 Promoting a healthy lifestyle is regarded as the most important strategy to prevent atherosclerotic cardiovascular disease (CVD).^1^ Healthy lifestyles have the potential to reduce CVD incidence by at least 50%.^2^ However, low and non-sustained adherence to lifestyle recommendations constitutes a major barrier to effective prevention.^3^

The initiation of CVD-preventive efforts in healthcare typically includes assessment and communication of CVD risk. This risk is often expressed in words or as a number.^4 5^ However, verbal or numerical information on risk alone seldom leads to an accurate risk perception that translates into a sustained lifestyle change and risk reduction.^6^ A promising way to improve the communication and perception of CVD risk is the use of carotid ultrasound. This enables visualisation of the underlying atherosclerosis itself, rather than just presenting the likelihood of future CVD.^7 8^

The Visualization of Asymptomatic Atherosclerotic Disease for Optimum Cardiovascular Prevention (VIPVIZA) is an ongoing randomised controlled trial conducted within a CVD prevention programme (the Västerbotten Intervention Programme (VIP)) in Swedish primary care. The programme consists of a health survey and a tailored health-promoting consultation with a trained nurse. In the intervention group, pictorial-based information about the participants’ carotid atherosclerosis was sent to the participants and their primary care physicians, while in the control group no such information was provided. Previous studies from the VIPVIZA project have demonstrated improved risk communication,^9^ increased prescription of and adherence to lipid-lowering medication,^10 11^ improvement of cardiovascular risk scores after 1 and 3 years of follow-up,^12 13^ reduced carotid intima-media thickness^14^ and greater accuracy in the participants’ perceptions of their CVD risk.^15^ However, it is unclear to what extent the enhanced risk perception provided by the pictorial-based information translates into healthier lifestyles.

The need for further studies on communication strategies to improve adherence to lifestyle recommendations in secondary prevention was emphasised in a recent consensus statement from the European Association of Preventive Cardiology.^16^ Evidence on the effects on behavioural outcomes of preventive efforts in primary care is likewise limited.^17^ Moreover, a systematic review and meta-analysis of the impact of pictorial information on different health behaviours concluded that the evidence for most outcomes was of low certainty.^18^ We therefore aimed to investigate the effect of pictorial-based information about carotid atherosclerosis provided in a primary care setting on adherence to lifestyle recommendations.

## Methods

### Design

VIPVIZA is a pragmatic, open-label randomised controlled trial conducted within an ongoing CVD prevention programme (the VIP) in Swedish primary care. VIPVIZA and VIP are described in detail elsewhere.^12 13 15 19 20^ In VIP, inhabitants in Västerbotten county (n=259 000 in 2013) in northern Sweden are invited to a health survey and health-promoting dialogue with a primary care nurse at the ages of 40, 50 and 60. The participation rate during 2007–2016 was 68%,^12^ and the selection bias was small.^21^ Before the start of the VIPVIZA trial, a pilot study was performed, and the intervention was calibrated according to participants’ experiences and suggestions collected through questionnaires and interviews.

### Participants

VIP participants from all healthcare centres in Västerbotten (covering an area of approximately 55.000 km², comparable to that of Croatia) were recruited to VIPVIZA with the following inclusion criteria:

1. 40 years of age and with a first-degree relative with CVD before the age of 60.
2. 50 years of age and with at least one CVD risk factor: smoking, diabetes, hypertension, hyperlipidaemia, waist circumference ≥102 cm (men) or ≥88 cm (women), or a first-degree relative with CVD before the age of 60.
3. 60 years of age.

Exclusion criteria were significant carotid stenosis (>50%) at baseline, violation of study protocol or participation in another clinical study during follow-up.

A total of 4177 individuals were invited by the nurse at their VIP visits, and 3532 were enrolled between 29 April 2013 and 7 June 2016.

### Intervention

At baseline, all individuals took part in the VIP intervention consisting of a questionnaire on CVD risk factors and lifestyle habits, a health examination and a tailored health-promoting dialogue with a trained nurse addressing diet, physical activity, alcohol consumption and tobacco use, adapted to each individual’s lifestyle habits. In addition, the VIPVIZA participants (intervention and control groups) underwent a carotid ultrasound examination performed by a biomedical technician according to a standardised protocol^14^ using portable ultrasound equipment. Atherosclerotic plaques and intima-media thickness were assessed on both sides, and the result was transformed into a pictorial presentation. At 1 and 3 years from baseline, the health examination and administration of the questionnaire were repeated for all study participants. In this study, we used data from baseline and the 3-year follow-up.

**Figure 1:**
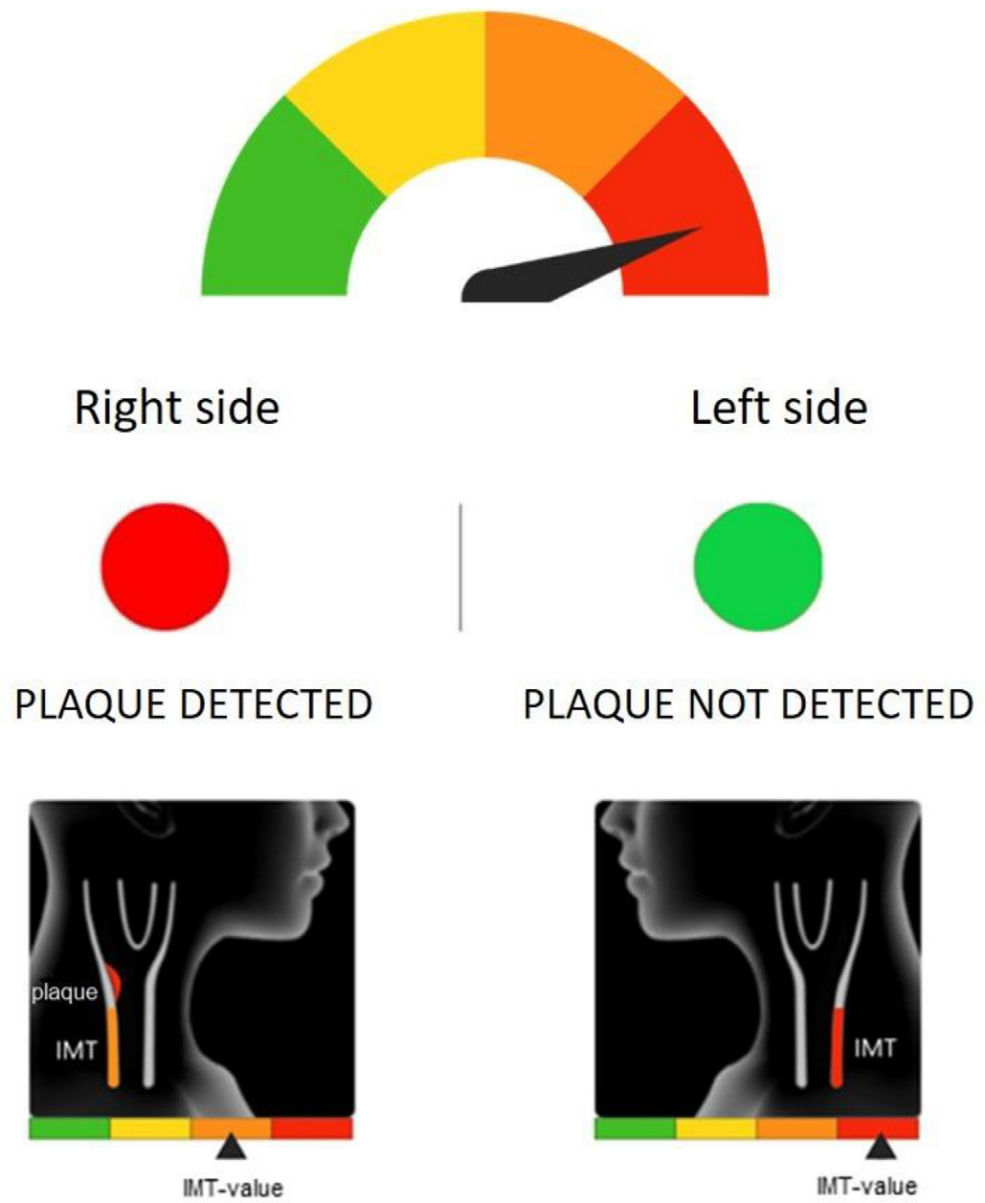
Pictorial information about individuals‘ carotid atherosclerosis provided in the intervention group. The gauge ranging from green through yellow and orange to red represents intima-media thickness (IMT) presented as vascular age from at least 10 years younger (green) to at least 10 years older (red). The presence of plaques is presented using a ‘traffic light’ system, with the absence of plaques indicated by a green dot and the presence of plaques indicated by a red dot.

The participants in the intervention group and their general practitioners received the pictorial information from the baseline carotid ultrasound examination by mail. In this report, intima-media thickness was presented on a colour-coded gauge ranging from green through yellow and orange to red, illustrating vascular age from at least 10 years younger to at least 10 years older. The presence of plaques was presented using a ‘traffic light’ system, with the absence of plaques indicated by a green dot and the presence of plaques indicated by a red dot (figure 1). Additionally, written information explaining atherosclerosis as a dynamic process that can be modified through lifestyle changes was provided. The complete written information to the intervention group is provided in online supplemental file 1.^15^ The participants received a follow-up phone call from a research nurse 2–4 weeks later. Any questions or concerns regarding the pictorial or written information were addressed, and the nurse engaged in a motivational dialogue about health-promoting lifestyle habits. At 6 months, the pictorial information was repeated to the participants, with a reminder of general preventive measures. At 9, 24 and 30 months, a letter was sent with information about the next follow-up visit, along with general information about healthy lifestyles and atherosclerosis. The intervention incorporates several behaviour change techniques, including biofeedback, information on health consequences and instructions on how to perform the behaviour (see online supplemental figure 1).^20^

In the control group, no pictorial or written information was provided, no follow-up calls were undertaken, no reminders were sent to the participants and no letters were sent to the general practitioners. Both groups received the VIP intervention and standard care.

### Outcomes

The primary outcome was a lifestyle index derived from questionnaire data covering the four major modifiable CVD risk factors—physical activity, smoking, alcohol consumption and diet, which are commonly included in lifestyle indices.^22^ The effects of these risk factors are interrelated and together account for a substantial proportion of CVD risk.^23 24^

Each of the lifestyle habits was scored on a scale of 1–3, with 3 indicating the most health-promoting behaviour. The lifestyle index was constructed as the sum of these scores and hence ranged from 4 to 12.

Physical activity was assessed from two questions:

Q1: ‘*To what extent have you been physically active during leisure time in the last 12 months?*’ (with four response alternatives): (1) low physical activity (less than 2 hours of activity per week), (2) moderate activity (at least 2 hours of low to moderate-intensity activity per week), (3) regular activity (high-intensity activity at least one to two times per week and at least 30 min per occasion) or (4) regular exercise (high-intensity exercise for a minimum of 30 min at least three times a week).

Q2: ‘*How much time do you spend during a regular week doing activities of moderate intensity that make you feel warm?*’ (with five response alternatives): (1) none or do not know, (2) less than 1 hour, (3) 1–3 hours, (4) 3–5 hours and (5) more than 5 hours.

Based on the responses to both questions, physical activity was scored as:

- 1 for a score of 1–2 on Q1 and 1–2 on Q2 (physically inactive).
- 2 for a score of 1–2 on Q1 and 3 on Q2, or a score of 3 on Q1 and 1–2 on Q2 (moderate physical activity up to 150 min/week).
- 3 for a score of 4 on Q1 (regardless of the score on Q2), or a score of 4–5 on Q2 (regardless of the score on Q1), or a score of 3 on both Q1 and Q2 (moderate physical activity more than 150 min/week or intense physical activity more than 75 min/week).

Smoking habits were assessed from a single question with the following response alternatives: (1) have never smoked, (2) current smoker of cigarettes, (3) current smoker of cigars, (4) current pipe smoker, (5) occasional smoker, (6) previous daily smoker and (7) previous occasional smoker. These answers were combined into an index where level 1 corresponded to current daily smoking (cigarettes, cigars or pipes), level 2 occasional smoking and level 3 never or previous smoking.

The alcohol consumption variable was calculated from the AUDIT (Alcohol Use Disorders Identification Test) questionnaire^25^ and scored as 1 for indication of alcohol dependency (≥16 points for men, ≥14 for women), 2 for indication of harmful alcohol consumption (8–15 points for men, 6–13 for women) and 3 for no indication of harmful consumption (≤7 points for men, ≤5 for women).

Diet was assessed based on a Food Frequency Questionnaire with 66 items.^26^ A Healthy Diet Score (HDS) of 0–24 was calculated from four favourable food groups (fish, fruit, vegetables and whole grains) and four unfavourable food groups (red and processed meat, desserts and sweets, sugar-sweetened beverages and fried potatoes).^27^ Intake frequencies were ranked within each sex for favourable foods in increasing quartile ranks (0, 1, 2, 3) and for unfavourable foods in decreasing quartile ranks (3, 2, 1, 0). A higher score corresponded to a healthier diet, reflecting key components of the Nordic Nutrition Recommendations. The distribution of the total HDS was categorised into three groups based on tertile cut-offs and scored as 1, 2 and 3 for the lower, middle and highest third, respectively.

There was a large number of missing values (506 in the intervention group and 478 in the control group) for the diet variable at 3 years from baseline, because the Food Frequency Questionnaire was not included in the 3-year follow-up routines until after about 12 months (in May 2017). We therefore also calculated an alternative lifestyle index where waist circumference was used as a proxy for the diet variable.^28^ Waist circumference was measured with a tape measure placed at the level between the lowest rib and the pelvic crest, with the subject in a standing position after a slight exhalation. Here, diet was scored as 1 for a circumference ≥102 cm (men) or ≥88 cm (women), 2 for a circumference of 94–101 cm (men) or 80–87 cm (women) and 3 for a circumference <94 cm (men) or <80 cm (women), according to the WHO expert consensus.^29^

### Sample size

A power analysis was performed before recruitment for the primary VIPVIZA study to ensure a probability of 80% to detect a true difference between groups at a significance level of 5%.^12^ The limiting outcome variable demanding the largest sample size was carotid intima-media thickness, and 3500 participants (assuming a dropout rate of 20%) were found to be sufficient. For the present substudy, no formal power analysis was performed.

### Randomisation and blinding

Participants were randomised 1:1 to the intervention group (n=1749) or control group (n=1783) using a computer-generated randomisation list prepared by a statistician before enrolment, with consecutive allocation of the participants by research nurses. The randomisation status was concealed from both the participants and the biomedical technicians when the baseline ultrasound examination was performed.

### Statistics

Baseline summary statistics are reported as numbers and proportions for categorical variables and as mean and SD for continuous variables.

Lifestyle index was treated as an ordinal outcome variable in the main analysis, avoiding any assumption of equidistance and distributional form. Accordingly, to assess the intervention effect on lifestyle index at the 3-year follow-up, we performed a complete case analysis using ordinal logistic regression controlling for baseline levels of lifestyle index (4–12, as a categorical variable) and additionally controlling for age (40, 50, 60 years), sex (male/female) and education level (basic, mid-level, high). We analysed potential treatment interactions for age, sex and education by including interaction terms in the regression model. Further, non-response analyses were performed with use of χ^2 ^tests, t-tests and ordinal regression to test for differences between individuals with missing data on diet at 3-year follow-up and individuals included in the main analysis, as well as between individuals with any missing data or lost to follow-up and those included in the main analysis. Additionally, we performed a sensitivity analysis where waist circumference was used as a proxy for the diet variable in an alternative lifestyle index. The VIPVIZA study protocol and the statistical analysis plan for this study are provided in online supplemental files 2 and 3 .

The analyses were performed with IBM SPSS Statistics V.28.

### Ethical statement

All participants provided their written informed consent. The study was registered at ClinicalTrials.gov (ref: NCT01849575).

## Results

**Figure 2:**
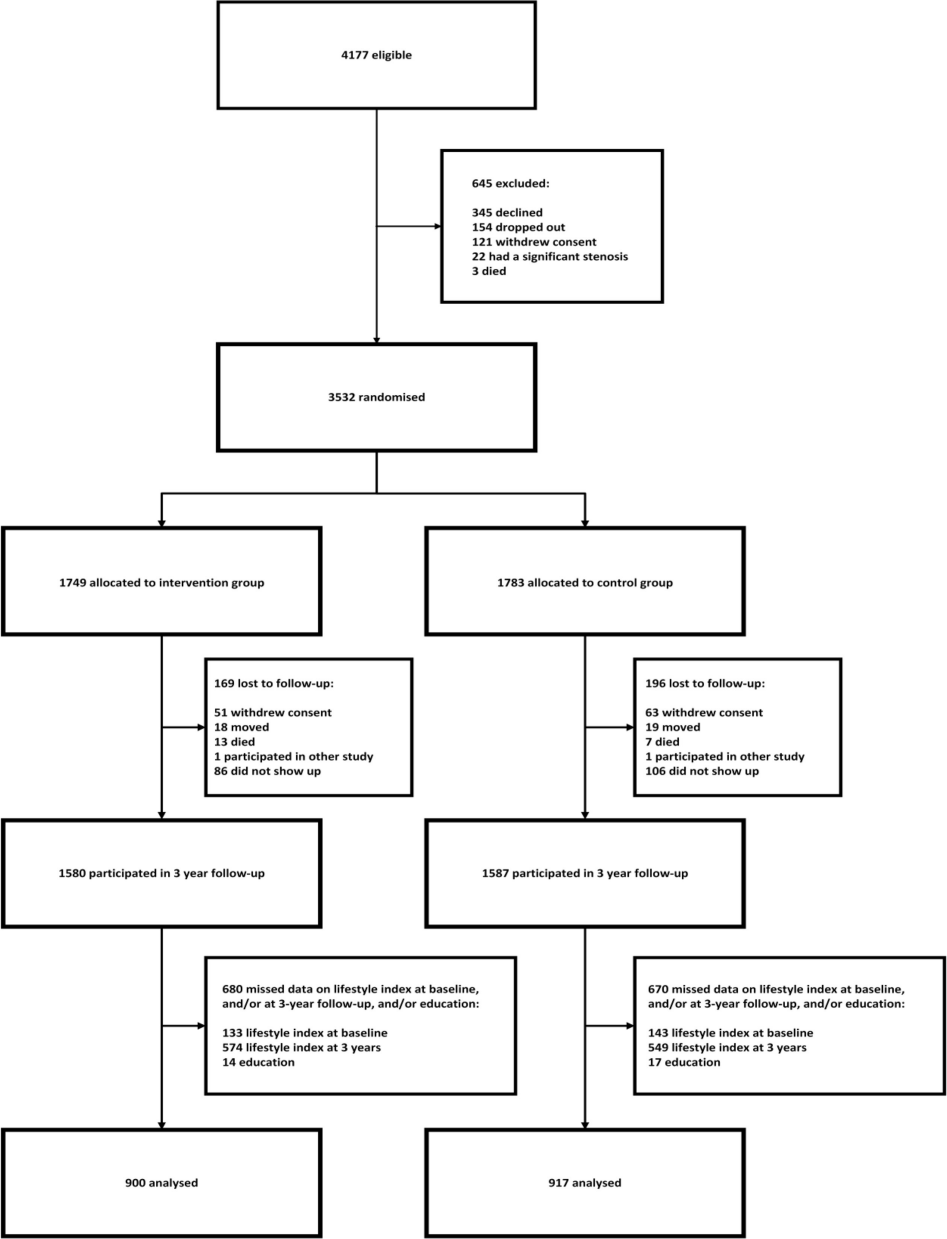
Flow chart of the participants included in the main analysis.

A total of 3167 individuals (1580 in the intervention group and 1587 in the control group) completed the 3-year follow-up. Of those individuals, 1817 with available data on education and lifestyle index at both baseline and the 3-year follow-up were included in the main analysis (900 in the intervention group and 917 in the control group). A flow chart of the study inclusion is presented in figure 2.

Baseline characteristics of the 1817 participants in the main analysis are presented in table 1, stratified by sex and randomisation group. At baseline, 1.8% of the participants had a lifestyle index of 4–6, 33.5% had an index of 7–9 and 64.7% had an index of 10–12.

**Table 1:** Baseline characteristics of the 1817 participants in the VIPVIZA study included in the main analysis

Characteristic	Men intervention (411)	Men control (418)	Women intervention (489)	Women control (499)	Total intervention (900)	Total control (917)
Age (years)						
40	28 (6.8)	30 (7.2)	27 (5.5)	38 (7.6)	55 (6.1)	68 (7.4)
50	99 (24.1)	121 (28.9)	146 (29.9)	122 (24.4)	245 (27.2)	243 (26.5)
60	284 (69.1)	267 (63.9)	316 (64.6)	339 (67.9)	600 (66.7)	606 (66.1)
Education*						
Basic	46 (11.2)	35 (8.4)	37 (7.6)	39 (7.8)	83 (9.2)	74 (8.1)
Mid-level	254 (61.8)	266 (63.6)	252 (51.5)	252 (50.5)	506 (56.2)	518 (56.5)
High	111 (27.0)	117 (28.0)	200 (40.9)	208 (41.7)	311 (34.6)	325 (35.4)
Physical activity†						
Low	68 (16.5)	73 (17.5)	76 (15.5)	81 (16.2)	144 (16.0)	154 (16.8)
Moderate	113 (27.5)	118 (28.2)	124 (25.4)	119 (23.8)	237 (26.3)	237 (25.8)
High	230 (56.0)	227 (54.3)	289 (59.1)	299 (59.9)	519 (57.7)	526 (57.4)
Diet (HDS)‡						
0-24	12.1 (3.5)	12.4 (3.6)	11.8 (3.7)	12.2 (3.9)	12.0 (3.6)	12.3 (3.8)
Alcohol consumption§						
Alcohol dependency	2 (0.5)	2 (0.5)	1 (0.2)	0 (0)	3 (0.3)	2 (0.2)
Risk consumption	49 (11.9)	34 (8.1)	19 (3.9)	35 (7.0)	68 (7.6)	69 (7.5)
Not at risk	360 (87.6)	382 (91.4)	469 (95.9)	464 (93.0)	829 (92.1)	846 (92.3)
Smoking						
Daily	37 (9.0)	28 (6.7)	42 (8.6)	48 (9.6)	79 (8.8)	76 (8.3)
Occasionally	11 (2.7)	18 (4.3)	14 (2.9)	17 (3.4)	35 (2.8)	35 (3.8)
Never/former	363 (88.3)	372 (89.0)	433 (88.5)	434 (87.0)	796 (88.4)	806 (87.9)
Waist (cm)						
>101/>87	153 (37.5)	175 (42.3)	282 (58.4)	294 (60.2)	435 (48.8)	469 (52.0)
94–101/80–87	154 (37.7)	143 (34.5)	115 (23.8)	110 (22.5)	269 (30.2)	253 (28.0)
<94/80	101 (24.8)	96 (23.2)	86 (17.8)	84 (17.2)	187 (21.0)	180 (20.0)
Lifestyle index¶						
4–6	9 (2.2)	9 (2.2)	6 (1.2)	8 (1.6)	15 (1.7)	17 (1.9)
7–9	143 (34.8)	139 (33.3)	157 (32.1)	170 (34.1)	300 (33.3)	309 (33.7)
10-12	259 (63.0)	270 (64.6)	326 (66.7)	321 (64.3)	585 (65.0)	591 (64.4)
Alternative lifestyle index**						
4–6	6 (1.5)	6 (1.4)	8 (1.7)	10 (2.0)	14 (1.6)	16 (1.8)
7–9	131 (32.1)	145 (35.0)	174 (36.0)	188 (38.5)	305 (34.2)	333 (36.9)
10-12	271 (66.4)	263 (63.5)	301 (62.3)	290 (59.4)	572 (64.2)	553 (61.3)

n (%) for categorical variables and mean (SD) for continuous variables.

* Basic: ≤9 years, compulsory level. Mid-level: 10–12 schooling years. High: ≥13 years, university level.

† Low: ≤60 min/week. Moderate: 60–150 min/week. High: ≥150 min/week.

‡ Calculated from questionnaire data on four favourable food groups and four unfavourable food groups.

§ Based on the AUDIT (Alcohol Use Disorder Identification Test) questionnaire. Alcohol dependency: ≥16 points (men), ≥14 points (women). Risk consumption: 8–15 points (men), 6–13 points (women). Not at risk: ≤7 points (men), ≤5 points (women).

¶ Represents the sum of scores (1–3) on physical activity, alcohol, smoking and diet (with HDS categorised into tertiles).

** Represents the sum of scores (1–3) on physical activity, alcohol, smoking and waist circumference.

HDS, Healthy Diet Score; VIPVIZA, Visualization of Asymptomatic Atherosclerotic Disease for Optimum Cardiovascular Prevention.

**Figure 3:**
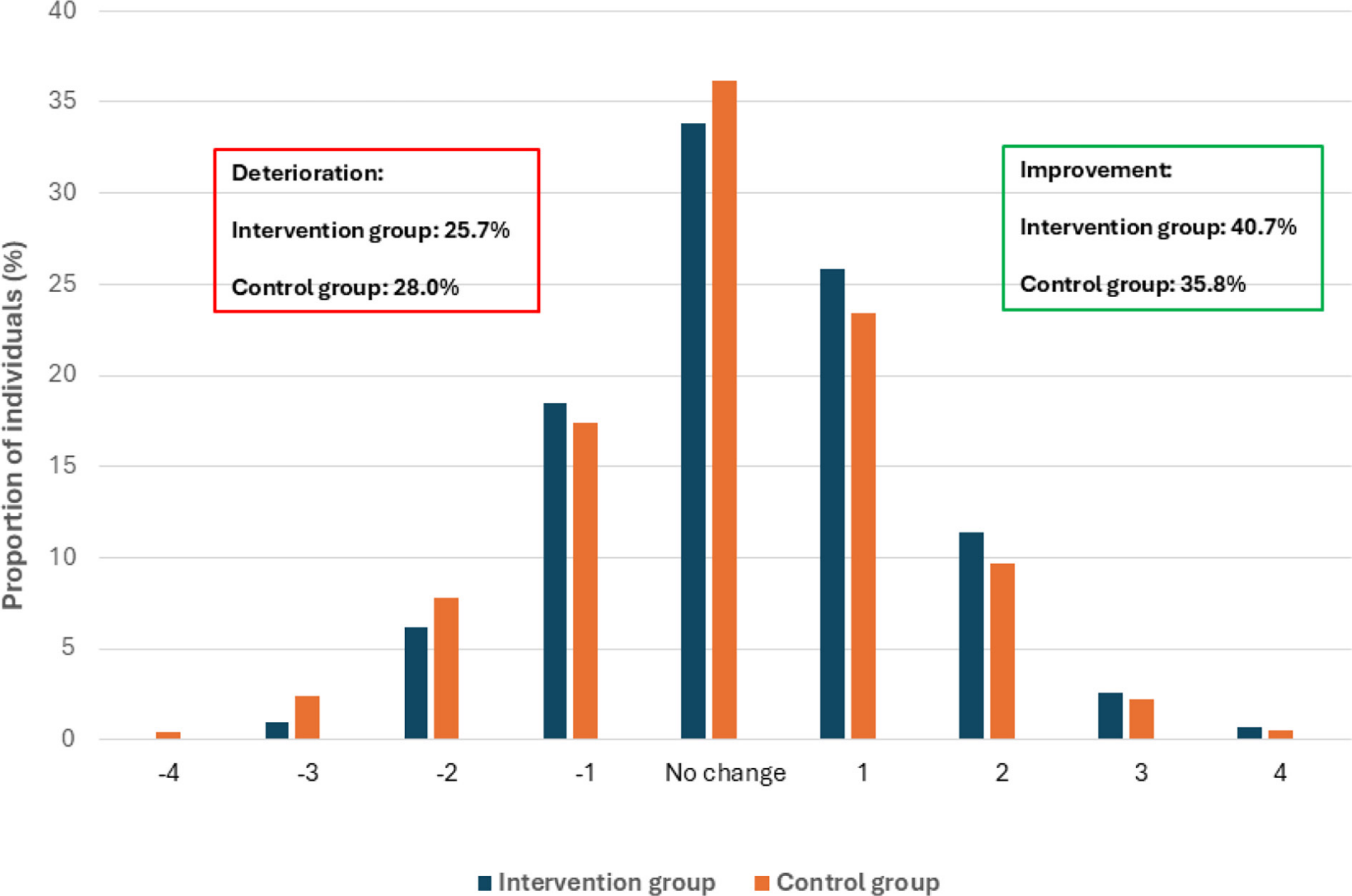
Change in lifestyle index from baseline to 3-year follow-up in the intervention group (blue bars) and control group (orange bars) ranging from −4 (deterioration) to +4 (improvement), with a summary of proportions of improvement and deterioration in each group.

The lifestyle index increased by 0.25 units from baseline to the 3-year follow-up in the intervention group and by 0.09 units in the control group. In the intervention group, 40.7% of the individuals increased their lifestyle index from baseline to the 3-year follow-up compared with 35.8% in the control group. A decline in lifestyle index occurred in 25.7% of participants in the intervention group and 28.0% in the control group (figure 3).

The OR of having a healthier lifestyle index for the intervention group versus the control group after 3 years, adjusted for baseline levels of lifestyle index, was 1.20 (95% CI 1.02 to 1.42, p=0.03) (table 2). The effect remained significant following additional adjustments for age, sex and education (OR 1.20, 95% CI 1.01 to 1.42, p=0.03). We found no significant interactions for age (p=0.64), sex (p=0.30) or education level (p=0.74).

**Table 2:** ORs for achieving a higher lifestyle index in the intervention group versus the control group

	Model 1* OR (95% CI) Participants in the analysis (n)	P value	Model 2† OR (95% CI) Participants in the analysis (n)	P value
Lifestyle index after 3 years‡	1.20 (1.02 to 1.42) 1829	0.03	1.20 (1.01 to 1.42) 1817	0.03
Alternative lifestyle index after 3 years§	1.15 (1.00 to 1.32) 2706	0.05	1.16 (1.01 to 1.33) 2687	0.04

* Model 1: adjusted for baseline levels of lifestyle index/alternative lifestyle index.

† Model 2: adjusted as in model 1 and additionally for age, sex and education level.

‡ Represents the sum of scores (1–3) on physical activity, alcohol consumption, smoking and diet (with HDS categorised into tertiles).

§ Represents the sum of scores (1–3) on physical activity, alcohol consumption, smoking and waist circumference.

HDS, Healthy Diet Score.

A total of 2687 individuals were included in the analysis with the alternative lifestyle index as outcome, where waist circumference was used as a proxy for diet. Baseline characteristics for those participants are presented in online supplemental table 1. In this sensitivity analysis, the OR for having a healthier lifestyle index was 1.15 (95% CI 1.00 to 1.32, p=0.05) for the intervention group versus the control group, in the model adjusted for baseline levels of the lifestyle index, and 1.16 (95% CI 1.01 to 1.33, p=0.04) in the model additionally adjusted for age, sex and education (table 2).

The proportions of participants across the different levels of the lifestyle indices at 3 years in the intervention and control groups are presented in online supplemental figure 2, together with similar figures for physical activity, diet and alcohol consumption. (Data on smoking and waist circumference have been published previously.^13^)

### Non-response analysis, dropouts and missing values

In the non-response analysis of baseline characteristics among individuals with missing data on diet at 3-year follow-up (n=984, the majority of whom did not receive the Food Frequency Questionnaire and thus were not assessed for diet at 3 years), persons with missing data differed significantly on HDS, waist circumference and lifestyle index from those included in the main analysis in the intervention group. No significant differences were found in any other baseline variables in either of the groups (online supplemental table 2).

The analysis of baseline characteristics among all study participants who were lost to follow-up for any reason (those who withdrew consent or did not show up at the 3-year follow-up, individuals lost to follow-up of other reasons and individuals with missing data, n=1715) demonstrated that persons lost to follow-up differed significantly from those in the main analysis on waist in both groups and on age, HDS and lifestyle index in the intervention group. No other significant differences in baseline variables were found (online supplemental table 3).

Proportions of missing data are presented in online supplemental table 4.

## Discussion

The main finding in this study was an improvement in a lifestyle index among 40.7% of the participants in the intervention group after receiving personalised, pictorial-based information on atherosclerosis, as compared with 35.8% in the control group. The effect was not moderated by age, sex or education. Notably, our intervention targeted a middle-aged study population with low-to-moderate CVD risk and a relatively healthy lifestyle at the outset and was conducted in addition to an ongoing CVD prevention programme with demonstrated cost-effectiveness^30^ and impact on cardiovascular mortality.^31^ This may have limited the potential for improvement due to ceiling effects, although the analytical method we used—ordinal regression—is considered relatively robust to such effects.^32^ From an epidemiological perspective, as the majority of CVD events occur in the large part of the population that are at moderate risk, focusing solely on high-risk individuals may overlook a significant potential for prevention.^33^ Furthermore, even small changes in lifestyle behaviours and risk factors, if sustained and adopted by many, will have significant effects on CVD incidence on a population level.^34^

Like many other efforts to convey graphic information about atherosclerosis, the VIPVIZA intervention should be considered multidimensional rather than strictly pictorial, since it consisted of an ultrasound examination followed by repeated pictorial and written information, in addition to verbal communication with a nurse. Several behaviour change techniques were used, such as providing biofeedback and feedback on health outcomes from a credible source, informing on and increasing salience about health consequences and instructing on how to perform different behaviours (online supplemental figure 1). The VIPVIZA intervention has been shown to enhance the accuracy of participants’ self-rated CVD risk without undermining their efficacy beliefs,^15^ both of which are important in promoting adherence to lifestyle recommendations.^16^ Furthermore, the interventions’ ability to evoke emotional arousal and cognitive engagement was positively associated with lifestyle change in the intervention group.^20^ Interestingly, strong emotional reactions, regardless of emotional valence, in combination with high levels of cognitive engagement, showed the strongest association with lifestyle change.^20^ These findings also suggest that different elements of the intervention (pictorial information, written information and dialogue with a nurse) contribute to cognitive and emotional responses to varying degrees; however, it remains difficult to disentangle how and to what extent each component influences outcomes. Given the multidimensional nature of the intervention, it is therefore not possible to determine precisely which aspects are responsible for the observed effects on lifestyle habits in our study.

To our knowledge, this is the first study to report results on a lifestyle index following a multicomponent intervention including pictorial information on subclinical atherosclerosis in a primary care context. Previous research on interventions in which individuals are provided with information about their CVD risk in conjunction with efforts to motivate lifestyle change has shown mixed or uncertain long-term results.^35^ In the recent TANSNIP (Trans-Atlantic Network to study Stepwise Non-invasive Imaging as a tool for CVD Prognosis and prevention) -PESA (Progression of Early Subclinical Atherosclerosis) randomised controlled trial, the intervention was comprehensive consisting of 12 motivational interviews over a period of 3 years, the use of a physical activity tracker and a sit-stand workstation.^36^ There was a significant effect on a compound outcome including physical activity, diet, tobacco use, weight and blood pressure after 1 year, but the effect was not sustained after 3 years, even though in contrast to our study, persons with healthier lifestyles and without atherosclerotic plaques were excluded in order to ensure sufficient room for improvement. It is worth noting that all participants (intervention+control groups) in the TANSNIP-PESA study received written (ie, not pictorial) information about their atherosclerosis status. The absence of the pictorial elements included in the VIPVIZA intervention, and the emotional and cognitive responses they may evoke, could partly explain why we observed sustained effect on lifestyle behaviours, whereas they did not. In contrast to the TANSNIP-PESA intervention, the CAUGHT-CAD (Coronary Artery calcium score: Use to Guide management of HerediTary Coronary Artery Disease) intervention incorporated pictorial information about atherosclerosis. Here, in total, 449 asymptomatic individuals aged 40–70 years with heredity for CVD were 1:1 randomised to a control group or an intervention consisting of repeated presentation of coronary artery calcium (CAC) scores and images to participants accompanied by motivational dialogues with a nurse. Additionally, all the participants in the intervention group received prescription of statins by the study team as well as antihypertensives in case of hypertension.^37 38^ The general practitioners were also informed about the participants’ CAC scores. At the 1-year follow-up, there was a significant difference in pooled cohort equation-based CVD risk in favour of the intervention group, but no effect on lifestyle habits was seen. Of the participants in the intervention group, 85% recalled their images, and 48% reported that the pictorial information had an impact on their adherence to therapy and recommendations. At the 3-year follow-up, a significant effect was noted both in Framingham Risk Score and physical activity. No significant effects on diet or smoking were seen, but interestingly, participants with sustained recall of their CAC images had a greater reduction in waist circumference when compared with those with unsustained recall. This highlights the potential importance of repeated exposure to images for enhanced recall and sustained effects, an approach that was also applied in our study.

Contradictory results have been seen from other studies evaluating specifically the use of carotid ultrasound-based pictorial information about atherosclerosis in order to influence lifestyle habits. Rodondi *et al* performed a randomised controlled trial with an intervention consisting of a carotid ultrasound examination in middle-aged smokers, followed by pictorial presentation of plaques and a 7-minute motivational dialogue, and found no significant reduction in smoking after 6 months.^39^ This might have been in part due to the intervention being too time limited and performed at one single time point only, in contrast to the VIPVIZA intervention. Repeated exposure to images was also included in the PreventiPlaque intervention described by Ullrich *et al*. It consisted of a mobile phone application with daily reminders on lifestyle recommendations during a period of 12 months, combined with a regularly updated pictorial presentation of ultrasound-generated pictures of individuals’ carotid plaques.^40^ The primary outcome, SCORE2 (Systematic COronary Risk Evaluation 2) risk, was significantly lower in the intervention group, but no effect on self-reported physical activity was demonstrated. This may be explained at least in part by the PreventiPlaque intervention not eliciting the same emotional and cognitive responses as the VIPVIZA intervention, as participants may become habituated to the daily reminders and ongoing interaction with the application. Identifying the optimal interval for repeated interventions in individuals may be challenging.

Importantly, we found no interaction with education in the current study. Several other trials have been conducted mainly in high-income and highly educated groups. For example, in a study by Rozanski *et al*,^41^ where CAC pictures were presented to the participants, 91% of the study population was highly educated; in the TANSNIP-PESA trial, 84% had a university degree as compared with 35% in our study sample. Since the prevalence of CVD is higher among persons with low education in Western countries,^42^ and the use of pictures to convey health information may be more efficient in people with low health literacy,^43^ our findings suggest that it is unlikely that the VIPVIZA intervention will contribute to increasing the social gap in health. However, interaction analyses generally have limited statistical power, and this should be considered when interpreting the results.

The limitations of this study include the use of self-reported lifestyle outcomes, which may introduce recall and social desirability bias as well as non-systematic misclassification. This may lead to attenuation of the observed effect and potentially to an underestimation of the true association, although this cannot be assumed with certainty.^44^ Preferably, lifestyle outcomes should be assessed by more objective measures such as accelerometers and biomarkers. However, the questionnaire included validated measures of diet and alcohol. Self-reported smoking has generally been shown to be valid, both in a Nordic and an international context.^45 46^ The physical activity questions were designed to reflect, as closely as possible, the items developed by the Swedish National Board of Health and Welfare, based on two measures capturing moderate and vigorous activities, previously validated by Kallings.^47^ We did not incorporate other CVD risk factors such as stress, sleep duration and social support in the lifestyle index, mainly because the intervention was not primarily directed towards these factors. Additionally, we did not apply different weights to the components in the index based on the relevance to the CVD risk of each lifestyle habit. Although weighting lifestyle habits based on their relative contribution to cardiovascular risk may appear more biologically plausible, such approaches introduce additional assumptions and complexity. Given the interrelated nature of lifestyle behaviours and the potential for measurement error in self-reported data, unweighted indices are often preferred for their robustness, transparency and usefulness. Moreover, the construction of a lifestyle index with ordinal categories can potentially result in an underestimation of the intervention effect, since changes within categories are neglected. The nature of our questionnaire data limited the possibilities of further subdivision into additional categories. At the same time, there is a limit to how small changes are clinically meaningful to detect. Another limitation of our study is the large number of participants who were lost to follow-up, mainly due to the substantial proportion of participants who were not assessed for the diet variable at the start of the 3-year follow-up. However, the results from the sensitivity analysis using a proxy variable for diet were consistent with those in the main analysis. The analyses of individuals who were lost to follow-up mainly for administrative reasons (due to the delayed administration of the Food Frequency Questionnaire at 3-year follow-up) or any reason showed minor differences in a small number of baseline characteristics, which are unlikely to have introduced significant bias. The lack of formal power analysis for this study is another limitation, which, in combination with the relatively large loss to follow-up, introduces a risk that the study was underpowered.

Our study has some strengths, among which are the population-based study sample with few exclusion criteria and a long follow-up period. The pragmatic design, with the trial incorporated within ordinary primary healthcare, and without involvement of the study team in the management of preventive measures in individuals, should provide high external validity, and suggests that the intervention could be readily implemented in many similar healthcare systems. Likewise, the ultrasound equipment is portable and accessible, easy to use even in rural areas and generates no irradiation. The different levels of our lifestyle index were largely based on clinically accepted and widely used cut-offs. The health-promoting dialogue in VIP and VIPVIZA is personalised, focusing on different lifestyle factors in each person. An evaluation of the intervention’s effect on each of the lifestyle factors separately could therefore potentially be misleading by underestimating the full impact of the intervention. Lifestyle indices offer a comprehensive, multidimensional approach considering the interrelations between different lifestyle factors.^22^

The intervention effect on adherence to healthy lifestyles is modest and borderline clinically relevant; however, when considered together with the previously demonstrated increase in statin use,^10 11^ it may be of clinical importance, as the combined adoption of a healthy lifestyle and statin therapy is associated with the greatest survival benefit.^48^ In order to determine whether the intervention will lead to a reduction in CVD mortality and morbidity, additional studies with extended follow-up are needed. Future research should also explore how personalised pictorial information about atherosclerosis could be further optimised to facilitate comprehension in different contexts, including among individuals with language difficulties and those with intellectual disabilities. The use of digital tools, including mobile apps, to support sustained lifestyle modification, as well as semiautomated carotid ultrasound equipment, should be further evaluated. Additional lifestyle outcomes such as sedentary behaviour, the use of new nicotine products, updated dietary intake, stress, social support and sleep should also be assessed, preferably measured by more objective methods.

## Conclusion

Our findings provide some further evidence for the use of ultrasound-based visualisation of subclinical carotid atherosclerosis, in conjunction with behavioural change techniques, to increase long-term adherence to a health-promoting lifestyle.^49^

## Supplementary material

10.1136/openhrt-2026-004136 online supplemental file 1

10.1136/openhrt-2026-004136 online supplemental file 2

10.1136/openhrt-2026-004136 online supplemental file 3

10.1136/openhrt-2026-004136 online supplemental figure 1

10.1136/openhrt-2026-004136 online supplemental figure 2

10.1136/openhrt-2026-004136 online supplemental table 1

10.1136/openhrt-2026-004136 online supplemental table 2

10.1136/openhrt-2026-004136 online supplemental table 3

10.1136/openhrt-2026-004136 online supplemental table 4

## Data Availability

Data are available upon reasonable request.
