# Supplementary material for: Effect of pictorial-based information about atherosclerosis on adherence to lifestyle recommendations: results from the VIPVIZA randomised controlled trial
Source: Open Heart. 2026 Jul 23;13(2):e004136. doi: 10.1136/openhrt-2026-004136 (PMC13404837; doi:10.1136/openhrt-2026-004136)
Supplement: online supplemental file 1 [file openhrt-13-2-s001.pdf]

Supplementary File 1.

Information to the participants in the intervention group

The general information under the heading "What can an ultrasound examination show?" is provided to all participants in the intervention group. Thereafter, personalized graphical information on plaque and IMT (based on the result of the ultrasound assessment) is presented. Explanations and recommendations are adapted to the participants' ultrasound results. A person with a favorable atherosclerosis status does not also read the advice aimed at a person with pronounced atherosclerosis. Participants with plaque, regardless of their IMT status, receive the red message. For participants without plaque, their IMT status (coded as green, yellow, orange, or red) determines which message they will receive.

WHAT CAN AN ULTRASOUND EXAMINATION SHOW?

The ultrasound examination measures two layers of the carotid artery wall (intima and media), the so-called IMT value.

A thickening of these layers is the first sign of atherosclerosis. Accumulation of calcium in the vessel wall, known as plaques, can also be detected and is a sign of more pronounced atherosclerosis, even if the wall thickness is not increased.

Atherosclerosis develops slowly from a young age, and is linked to lifestyle, heredity, blood pressure, blood lipids, blood sugar and weight. This was measured and discussed with you at your health check-up.

Atherosclerosis increases the risk of heart attack, stroke and shortened life expectancy due to cardiovascular disease. On the positive side, the development of atherosclerosis is a dynamic process that you can influence and thus also your risk of disease.

Atherosclerosis can be slowed down and even reversed.

This is accomplished through a healthy lifestyle including being physically active and eating healthy, and avoiding smoking and stress.

If you are prescribed medication for high blood pressure, high blood lipids or diabetes, it is important to take your medication in order for the treatment to be effective.

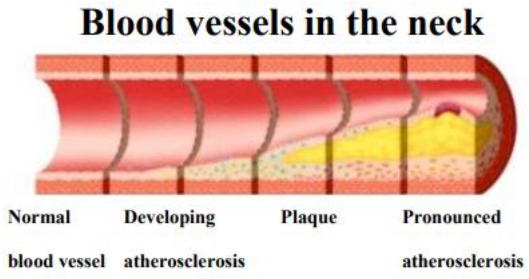

NOTE! This is not your blood vessel, but a schematic picture of how atherosclerosis can develop over time.

People with severe constriction of the blood vessels who need urgent treatment are referred for further investigation, and do not receive this message. You do not belong to this group.

Personal pictorial information provided here.

On the last page, based on the results of your ultrasound examination, there are recommendations on what you can do to promote your own health.

In people with any risk factor for cardiovascular disease (such as smoking, diabetes, high blood lipids or family history of cardiovascular disease), plaque is:

- uncommon at the age of 40,

- present in 10-15% at age 50 and

- present in 30-40%, i.e. quite common, at the age of 60.

WHAT CAN YOU DO YOURSELF TO STAY HEALTHY?

The ultrasound examination of your carotid arteries shows the thickness of the vessel wall and whether there are plaques. What to do is determined by the most pronounced change in your vessels. If you have a thickened vessel wall and/or plaque on one side, this will determine what to do, even if there are less pronounced changes on the other side.

THE ULTRASOUND EXAMINATION OF YOUR CAROTID ARTERIES CAN BE SUMMARISED AS FOLLOWS:

The color chart takes your age into account.

**Green**	No or less pronounced signs of atherosclerosis.<br>It is important **TO MAINTAIN A HEALTHY LIFESTYLE.**
**Yellow**	Moderate signs of atherosclerosis.<br>Yellow is <u>below</u> the average for your age.<br>It is important **TO SLOW DOWN OR REVERSE THE DEVELOPMENT OF ATHEROSCLEROSIS THROUGH A HEALTHY LIFESTYLE.**
**Orange**	Moderate signs of atherosclerosis.<br>Orange is <u>above</u> the average for your age.<br>It is important **TO SLOW DOWN OR REVERSE THE DEVELOPMENT OF ATHEROSCLEROSIS THROUGH A HEALTHY LIFESTYLE.**
**Red**	Pronounced signs of atherosclerosis.<br>It is important **TO SLOW DOWN OR REVERSE THE DEVELOPMENT OF ATHEROSCLEROSIS THROUGH A HEALTHY LIFESTYLE.**

REGARDLESS OF OBTAINED COLOUR SIGNAL, FOR INDIVIDUALS TAKING MEDICATION FOR:

- HIGH BLOOD LIPIDS

- HIGH BLOOD PRESSURE or

- DIABETES

It is important also to **TAKE YOUR MEDICATION**, in order for the medical treatment to be effective.

USEFUL INFORMATION TO BE FOUND ON THE INTERNET

The following links will provide you with a lot of good information and also additional links about healthy lifestyles and tips and support for changing your habits.

http://www.sundkurs.se

http://www.halsa2020.se

Reference:

Andersson EM, Liv P, Nordin S, et al. Does a multi-component intervention including pictorial risk communication about subclinical atherosclerosis improve perceptions of cardiovascular disease risk without deteriorating efficacy beliefs? Soc Sci Med. 2024;341:116530. https://doi.org/10.1016/j.socscimed.2023.116530
