# Supplementary material for: Effect of pictorial-based information about atherosclerosis on adherence to lifestyle recommendations: results from the VIPVIZA randomised controlled trial
Source: Open Heart. 2026 Jul 23;13(2):e004136. doi: 10.1136/openhrt-2026-004136 (PMC13404837; doi:10.1136/openhrt-2026-004136)
Supplement: online supplemental file 3 [file openhrt-13-2-s003.pdf]

Supplementary File 3.

Statistical Analysis Plan

Version Date 2021-11-29

Aims and objectives

Objective: to evaluate the effect of the VIPVIZA-intervention on lifestyle habits.

We aim to study

1. The association at baseline, in the whole study population (i.e. the intervention and the control group), between lifestyle index and carotid atherosclerosis (IMT and presence of plaque).

2. Lifestyle index in the intervention group and the control group at 3-year follow-up.

Population

The VIPVIZA participants (intervention and control group) at baseline and at 3-year follow-up.

Exposure/intervention/prognostic factor

For research question 1 the exposure (independent variable) is lifestyle index.

For research question 2 the intervention is the VIPVIZA intervention

Descriptive statistics to be presented

Descriptive statistics at baseline will be presented for the intervention and control group, and for men and women separately.

Continuous variables will be presented as means, and categorical variables as number of participants and percentages.

FRS

SCORE

Plaque (no, one side, both sides)

IMT

Age

Sex

P-Total Cholesterol

P-LDL

P-HDL

P-triglycerides

Systolic BP

Diastolic BP

Fp-glucose

Weight

Waist circumference

Education (low/medium/high)

Lifestyle index (4-12 or according to the updated lifestyle index)

Healthy diet score (1-3)

Physical activity (1-3)

Alcohol (1-3 and/or according to variable alc4_0)

Smoking (1-3)

Cross-sectional association analyses

For research question 1, linear regression will be used with IMT as dependent variable and lifestyle index as independent variable. For the analyses of plaque presence, ordinal logistic regression will be used with lifestyle index as independent variable.

Primary analyses model

For research question 2, ordinal logistic regression will be used. Dependent variable will be lifestyle index at 3 year follow up. VIPVIZA intervention group will the independent variable.

Due to large proportion of missing data on diet at the 3 year follow up, we will use waist circumference as proxy for diet in the primary analysis.

Shape of the association

Primarily, the analysis will be based on categorized exposure variables. The shape of the associations in research question 1 will be graphically visualized using scatter plots and forest plots.

Pre-specific subgroup analyses

Education (low, medium, high)

Sex (M/F)

Age group (40, 50, 60)

Lifestyle index at baseline

Secondary/sensitivity analyses

As a secondary analysis we will use a restricted dataset (n= 2044) with data on diet at baseline and at 3 year follow up.

Approach to deal with missing data

Patterns of missing data will be investigated and reported in paper. Multiple imputation will be performed as a sensitivity analysis of potential bias due to dropout and missing values. Results from the multiple imputed analyses of all analyses, respectively, will be pooled and presented.

Multiple testing

No adjustments for multiple testing are planned. Increased risk of familywise error due to multiple significance testing will be informally accounted for when discussing the interpretation of the results.

Assessment of uncertainties of estimated parameters

For all effect estimates, 95% confidence interval will be estimated.

Adjustment for confounding

Research question 1 will be adjusted for

Model 1: age, sex, education

Model 2: model 1 + potential mediators (LDL-cholesterol, systolic blood pressure, antihypertensive treatment, lipid-lowering treatment and diabetes)

Research question 2 will be adjusted for lifestyle index at baseline.

Statistical power estimate

Per Liv, statistician within the VIPVIZA project, will supervise and contribute to the analyses.

No formal power analysis has been conducted. However, a preliminary descriptive analysis showes that out of 3167 participants with data from baseline and the 3y follow-up, more than 350 participants have increased their lifestyle score with 2 or more and 670 participants have decreased their score with 2 or more after 3 years.
