# Supplementary material for: Effect of pictorial-based information about atherosclerosis on adherence to lifestyle recommendations: results from the VIPVIZA randomised controlled trial
Source: Open Heart. 2026 Jul 23;13(2):e004136. doi: 10.1136/openhrt-2026-004136 (PMC13404837; doi:10.1136/openhrt-2026-004136)
Supplement: online supplemental figure 1 [file openhrt-13-2-s004.pdf]

Supplementary Figure 1.

Behavioural change techniques in the VIPVIZA intervention.

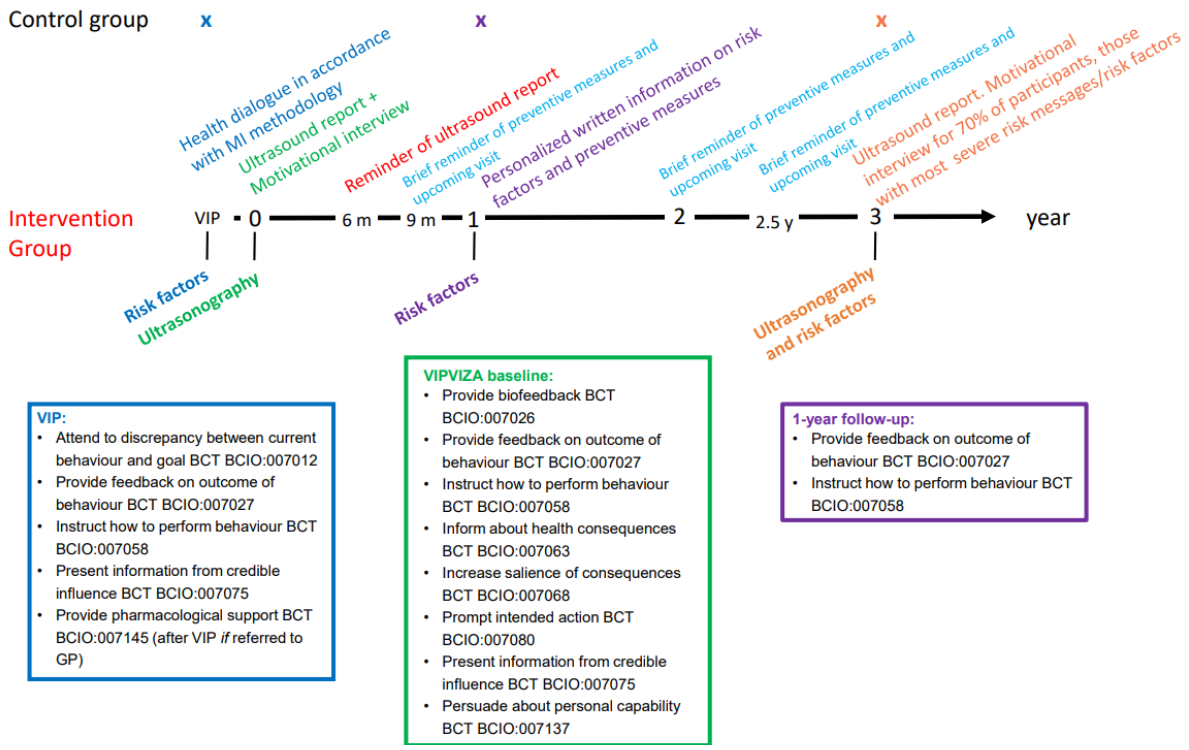

Reference:

Andersson EM, Lindvall K, Wennberg P, et al. From risk communication about asymptomatic atherosclerosis to cognitive and emotional reactions and lifestyle modification. BMC Psychol. 2024;12(1):47. https://doi.org/10.1186/s40359-023-01467-x
