# Supplementary material for: Effect of pictorial-based information about atherosclerosis on adherence to lifestyle recommendations: results from the VIPVIZA randomised controlled trial
Source: Open Heart. 2026 Jul 23;13(2):e004136. doi: 10.1136/openhrt-2026-004136 (PMC13404837; doi:10.1136/openhrt-2026-004136)
Supplement: online supplemental figure 2 [file openhrt-13-2-s005.pdf]

Supplementary Figure 2.

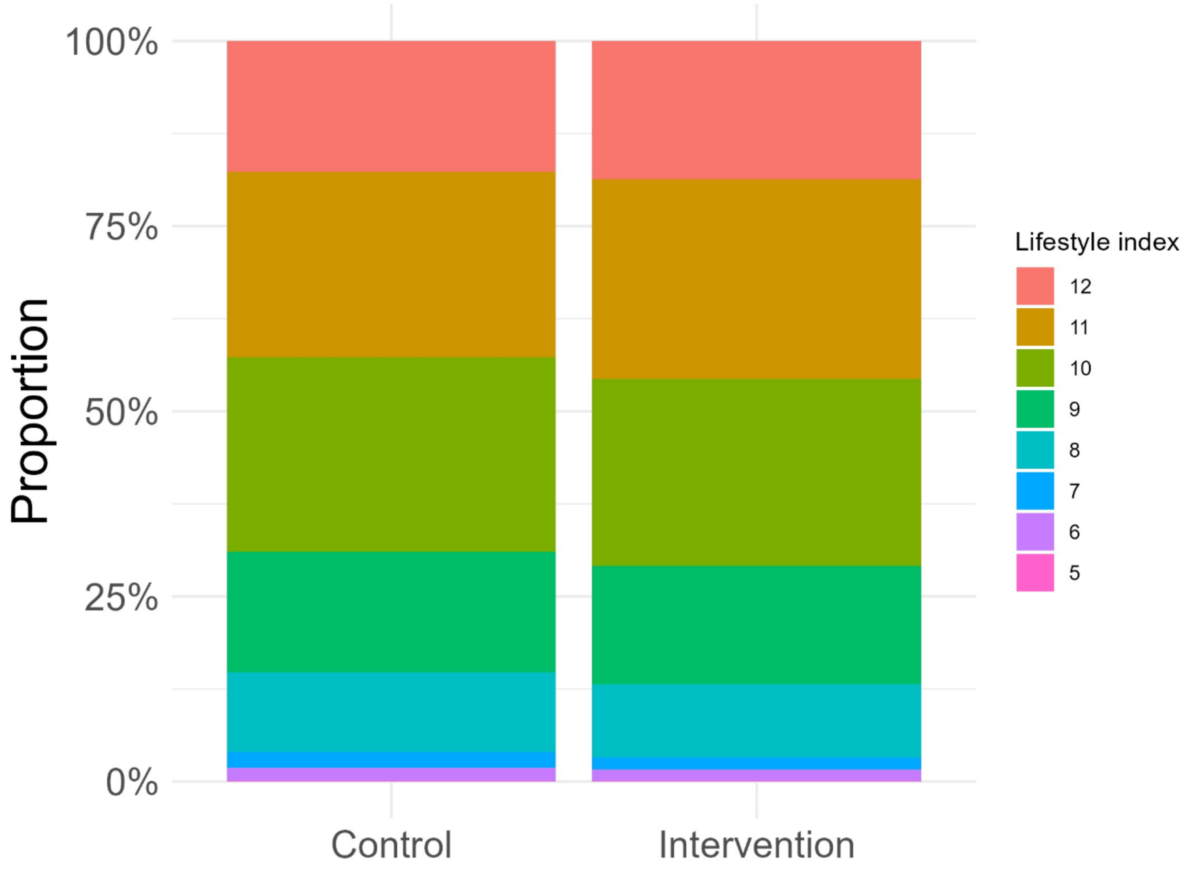

1. Proportion of participants across different levels of Lifestyle Index at 3-year follow-up in the control (n=922) and intervention (n=907) groups.

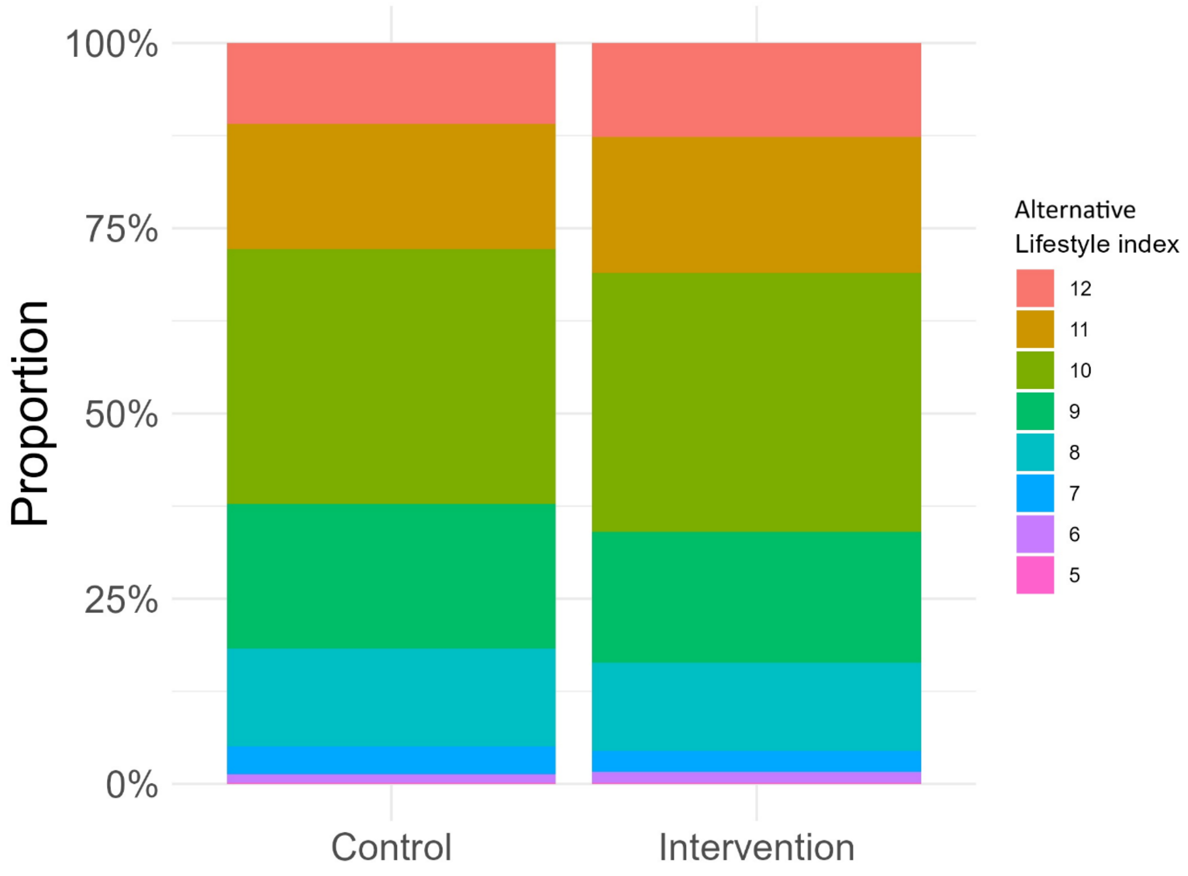

2. Proportion of participants across different levels of the Alternative Lifestyle Index at 3-year follow-up in the control (n=1349) and intervention (n=1357) groups.

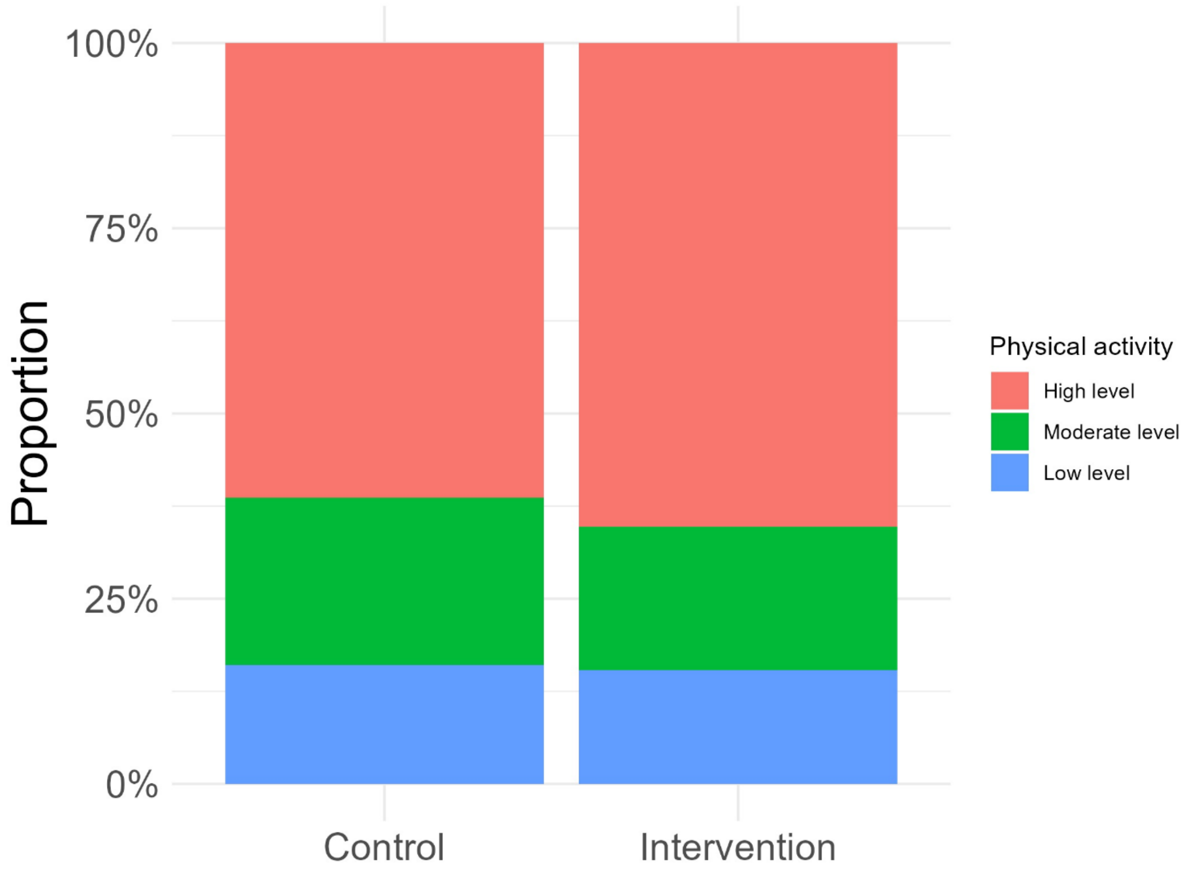

3. Proportion of participants across different levels of Physical Activity at 3-year follow-up in the control (n=1349) and intervention (n=1357) groups.

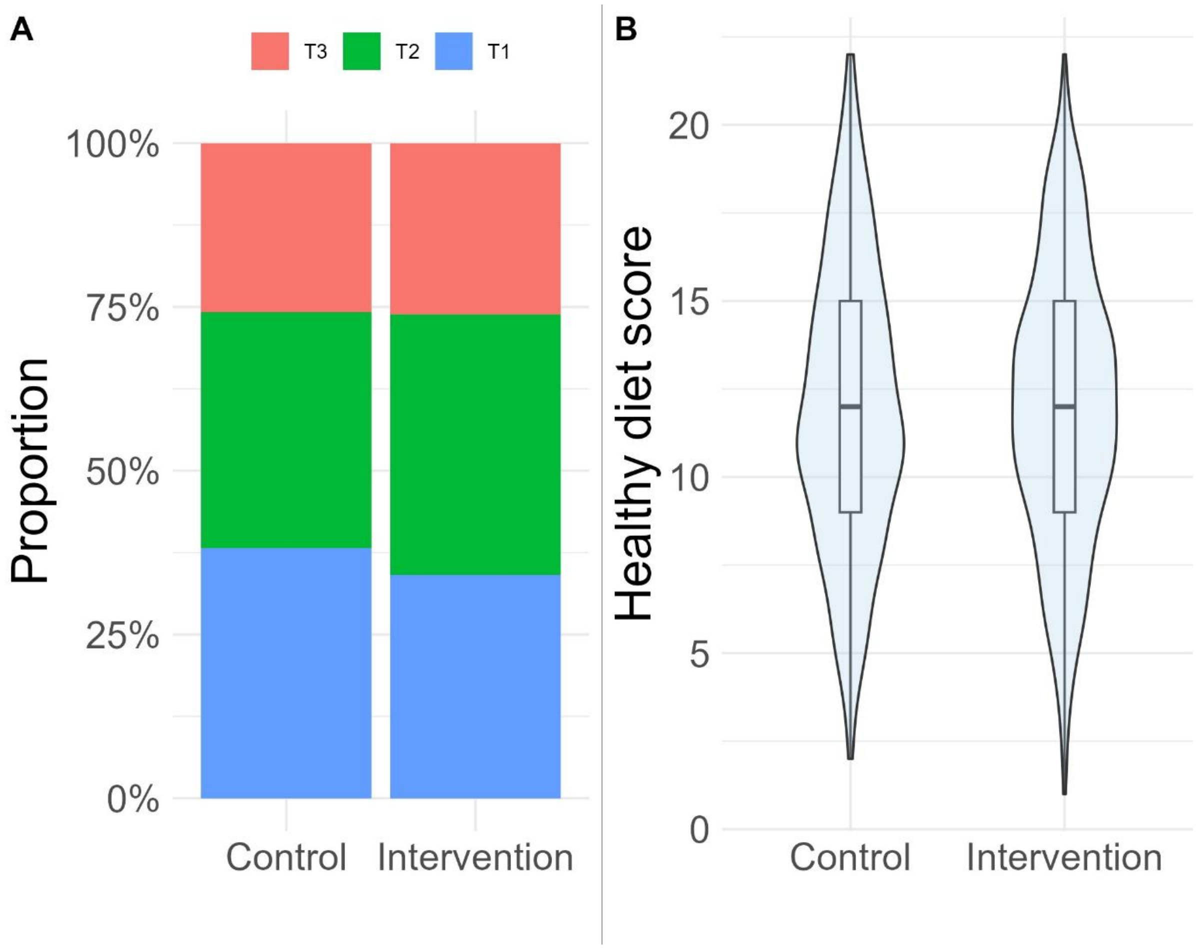

4. Healthy Diet Score in control (n=922) and intervention (n=907) groups at 3-year follow-up.
(A) Proportion of participants in the control and intervention groups across tertiles of the Healthy Diet Score. Tertile cutoffs were defined as T1 (lowest third), T2 (middle third), and T3 (highest third).
(B) Healthy Diet Score presented as a continuous variable (range 0–24) in the control and intervention groups at 3-year follow-up. The violin plot (light blue) illustrates the distribution of the data. The embedded boxplot shows the median (central line), interquartile range (box), and whiskers showing min and max values.

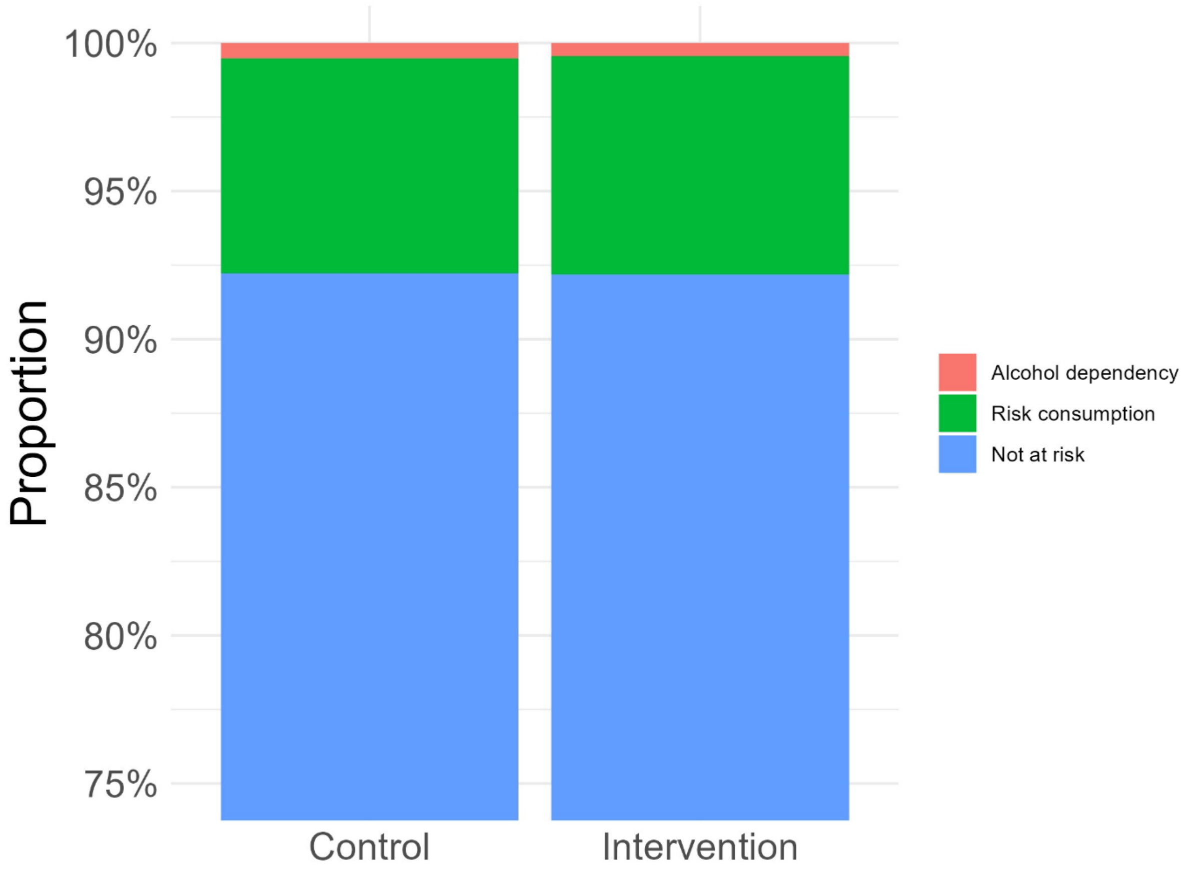

5. Proportion of participants across different levels of Alcohol Consumption at 3-year follow-up in the control (n=1349) and intervention (n=1357) groups. The y-axis is truncated (75–100%) to improve visualization.
