# Supplementary material for: Effect of pictorial-based information about atherosclerosis on adherence to lifestyle recommendations: results from the VIPVIZA randomised controlled trial
Source: Open Heart. 2026 Jul 23;13(2):e004136. doi: 10.1136/openhrt-2026-004136 (PMC13404837; doi:10.1136/openhrt-2026-004136)
Supplement: online supplemental table 1 [file openhrt-13-2-s006.pdf]

Supplementary table 1. Baseline characteristics of the 2687 participants analysed with the alternative lifestyle index as outcome variable.

	**Men** n=1250 (46.5)		**Women** n=1437 (53.5)		**Total** n=2687	
	**Intervention** 622 (49.8)	**Control** 628 (50.2)	**Intervention** 726 (50.5)	**Control** 711 (49.5)	**Intervention** n=1348 (50.2)	**Control** n=1339 (49.8)
**Age (years)**						
40	48 (7.7)	51 (8.1)	49 (6.7)	52 (7.3)	97 (7.2)	103 (7.7)
50	168 (27.0)	177 (28.2)	209 (28.8)	171 (24.1)	377 (28.0)	348 (26.0)
60	406 (65.3)	400 (63.7)	468 (64.5)	488 (68.6)	874 (64.8)	888 (66.3)
**Education**[1]						
Basic	57 (9.2)	55 (8.8)	57 (7.9)	55 (7.7)	114 (8.5)	110 (8.2)
Mid-level	383 (61.6)	384 (61.1)	378 (52.1)	352 (49.5)	761 (56.5)	736 (55.0)
High	182 (29.3)	189 (30.1)	291 (40.1)	304 (42.8)	473 (35.1)	493 (36.8)
**Physical activity**[2]						
Low	108 (17.4)	114 (18.2)	114 (15.7)	107 (15.0)	222 (16.5)	221 (16.5)
Moderate	166 (26.7)	170 (27.1)	182 (25.1)	177 (24.9)	348 (25.8)	347 (25.9)
High	348 (55.9)	344 (54.8)	430 (59.2)	427 (60.1)	778 (57.7)	771 (57.6)
**Diet (healthy diet score)**[3] 0-24	12.5 (3.5)	12.3 (3.6)	12.0 (3.7)	12.3 (3.9)	12.2 (3.6)	12.3 (3.8)
**Alcohol consumption**[4]						
Alc. dependency	2 (0.3)	5 (0.8)	4 (0.6)	0 (0)	6 (0.4)	5 (0.4)
Risk consumption	65 (10.5)	47 (7.5)	29 (4.0)	52 (7.3)	94 (7.0)	99 (7.4)
Not at risk	555 (89.2)	576 (91.7)	693 (95.5)	659 (92.7)	1248 (92.6)	1235 (92.2)
**Smoking**						
Daily	43 (6.9)	44 (7.0)	65 (9.0)	66 (9.3)	108 (8.0)	110 (8.2)
Occasionally	19 (3.1)	29 (4.6)	17 (2.3)	27 (3.8)	36 (2.7)	56 (4.2)
Never/former	560 (90.0)	555 (88.4)	644 (88.7)	618 (86.9)	1204 (89.3)	1173 (87.6)
**Waist (cm) m/f**						
>101/>87	257 (41.3)	265 (42.2)	430 (59.2)	436 (61.3)	687 (51.0)	701 (52.4)
94-101/80-87	194 (31.2)	207 (33.0)	165 (22.7)	151 (21.2)	359 (26.6)	358 (26.7)
<94/80	171 (27.5)	156 (24.8)	131 (18.0)	124 (17.4)	302 (22.4)	280 (20.9)
**Lifestyle index**[5]						
4-6	9 (1.5)	15 (2.5)	11 (1.6)	12 (1.8)	20 (1.6)	27 (2.2)
7-9	192 (32.9)	192 (32.4)	210 (30.3)	224 (33.8)	402 (31.5)	416 (33.1)
10-12	383 (65.6)	385 (65.0)	471 (68.1)	427 (64.4)	854 (66.9)	812 (64.7)
**Alternative lifestyle index**[6]						
4-6	9 (1.4)	9 (1.4)	16 (2.2)	16 (2.3)	25 (1.9)	25 (1.9)
7-9	201 (32.3)	218 (34.7)	257 (35.4)	269 (37.8)	458 (34.0)	487 (36.4)
10-12	412 (66.2)	401 (63.9)	453 (62.4)	426 (59.9)	865 (64.2)	827 (61.8)

n (%) for categorical variables and mean (SD) for continuous variables

[1] Basic: ≤9 years, compulsory level, mid-level: 10-12 schooling years, high: ≥13 years, university level
[2] Low: ≤60 minutes/week, moderate: 60-150 minutes/week, high: ≥150 minutes/week
[3]Calculated from questionnaire-data on four favourable food groups and four unfavourable food groups
[4]Based on the AUDIT questionnaire. Alcohol dependency: ≥16p (men), ≥14p (women), risk consumption: 8–15p (men), 6–13p (women), not at risk: ≤7p (men), ≤5p (women)
[5]Represents the sum of scores (1-3) on physical activity, alcohol, smoking and diet (with HDS categorized into tertiles)
[6]Represents the sum of scores (1-3) on physical activity, alcohol, smoking and waist
