# Supplementary material for: Effect of pictorial-based information about atherosclerosis on adherence to lifestyle recommendations: results from the VIPVIZA randomised controlled trial
Source: Open Heart. 2026 Jul 23;13(2):e004136. doi: 10.1136/openhrt-2026-004136 (PMC13404837; doi:10.1136/openhrt-2026-004136)
Supplement: online supplemental table 2 [file openhrt-13-2-s007.pdf]

Supplementary table 2. Non-response analysis of individuals with missing data on diet at 3-year follow-up, with respect to baseline characteristics.

	Intervention			Control		
	No healthy diet score at 3-year follow-up n=506	Complete data on lifestyle index and education n=900	p	No healthy diet score at 3-year follow-up n=478	Complete data on lifestyle index and education n=917	p
**Sex**						
Men	237 (46.8)	411 (45.7)	0.71	241 (50.4)	418 (45.6)	0.10
Women	269 (53.2)	489 (54.3)		237 (49.6)	499 (54.4)	
**Age (years)**						
40	48 (9.5)	55 (6.1)	0.06	41 (8.6)	68 (7.4)	0.73
50	139 (27.5)	245 (27.2)		123 (25.7)	243 (26.5)	
60	319 (63.0)	600 (66.7)		314 (65.7)	606 (66.1)	
**Education[1]**						
Basic	39 (7.7)	83 (9.2)	0.45	40 (8.4)	74 (8.1)	0.12
Mid-level	277 (55.0)	506 (56.2)		241 (50.8)	518 (56.5)	
High	188 (37.3)	311 (34.6)		193 (40.7)	325 (35.4)	
**Physical activity[2]**						
Low	87 (17.5)	144 (16.0)	0.71	85 (17.9)	154 (16.8)	0.87
Moderate	124 (24.9)	237 (26.3)		123 (25.8)	237 (25.8)	
High	286 (57.5)	519 (57.7)		268 (56.3)	526 (57.4)	
**Healthy diet score[3]** 0-24	12.7 (3.8)	12.0 (3.6)	**<0.001**	12.3 (3.8)	12.3 (3.8)	0.95
**Alcohol consumption[4]**						
Alc dependency	3 (0.6)	3 (0.3)	0.57	1 (0.2)	2 (0.2%)	0.74
Risk consumpt.	32 (6.5)	68 (7.6)		41 (8.7)	69 (7.5)	
Not at risk	460 (92.9)	829 (92.1)		428 (91.1)	846 (92.3)	
**Smoking**						
Daily	39 (7.7)	79 (8.8)	0.73	40 (8.4)	76 (8.3)	0.45
Occasionally	16 (3.2)	25 (2.8)		25 (5.3)	35 (3.8)	
Never/former	451 (89.1)	796 (88.4)		411 (86.3)	806 (87.9)	
**Waist (cm) m/f**						
>101/87	274 (54.6)	435 (48.8)	**<0.001**	243 (51.9)	469 (52.0)	0.29
94-101/80-87	101 (20.1)	269 (30.2)		117 (25.0)	253 (28.0)	
<94/80	127 (25.3)	187 (21.0)		108 (23.1)	180 (20.0)	
**Lifestyle index[5]**						
Group with available data	**Crude: OR 1.23 (95% CI 1.01-1.49), p=0.04**			Crude: OR 0.94 (95% CI 0.77-1.14), p=0.51		

as reference category	Model 1: OR 1.21 (95% CI 1.00-1.48), p=0.06	Model 1: OR 0.91 (95% CI 0.74-1.11), p=0.33
**Alternative lifestyle index**[6]		
Group with available data as reference category	Crude: OR 0.96 (95% CI 0.79-1.17), p=0.66<br><br>Model 1: OR 0.92 (95% CI 0.76-1.12), p=0.41	Crude: OR 0.98 (95% CI 0.80-1.20), p=0.85<br><br>Model 1: OR 0.92 (95% CI 0.75-1.12), p=0.41

n (%) or mean (SD), Chi2-test for categorical variables, T-test for continuous variables and ordinal regression for lifestyle index.
Model 1: Adjusted for age, sex, and education
[1] Basic: ≤9 years, compulsory level, mid-level: 10-12 schooling years, high: ≥13 years, university level
[2] Low: ≤60 minutes/week, moderate: 60-150 minutes/week, high: ≥150 minutes/week
[3]Calculated from questionnaire-data on four favourable food groups and four unfavourable food groups
[4]Based on the AUDIT questionnaire. Alcohol dependency: ≥16p (men), ≥14p (women), risk consumption: 8–15p (men), 6–13p (women), not at risk: ≤7p (men), ≤5p (women)
[5]Represents the sum of scores (1-3) on physical activity, alcohol, smoking and diet (with HDS categorized into tertiles)
[6]Represents the sum of scores (1-3) on physical activity, alcohol, smoking and waist
