# Supplementary material for: Effect of pictorial-based information about atherosclerosis on adherence to lifestyle recommendations: results from the VIPVIZA randomised controlled trial
Source: Open Heart. 2026 Jul 23;13(2):e004136. doi: 10.1136/openhrt-2026-004136 (PMC13404837; doi:10.1136/openhrt-2026-004136)
Supplement: online supplemental table 3 [file openhrt-13-2-s008.pdf]

Supplementary table 3. Analysis of all individuals without complete data at 3-year follow-up (missing data, lost to follow-up, dropouts), with respect to baseline characteristics.

	Intervention group			Control group		
	Missing data, dropouts and lost to follow-up n=849	Available data n=900	p	Missing data, dropouts and lost to follow-up n=866	Available data n=917	p
**Sex**						
Men	398 (46.9)	411 (45.7)	0.65	435 (50.2)	418 (45.6)	0.06
Women	451 (53.1)	489 54.3)		431 (49.8)	499 (54.4)	
**Age (years)**						
40	79 (9.3)	55 (6.1)	**0.03**	74 (8.5)	68 (7.4)	0.31
50	241 (28.4)	245 (27.2)		249 (28.8)	243 (26.5)	
60	529 (62.3)	600 (66.7)		543 (62.7)	606 (66.1)	
**Education[1]**						
Basic	83 (9.9)	83 (9.2)	0.84	83 (9.8)	74 (8.1)	0.41
Mid-level	471 (56.4)	506 (56.2)		462 (54.7)	518 (56.5)	
High	281 (33.7)	311 (34.6)		300 (35.5)	325 (35.4)	
**Physical activity[2]**						
Low	151 (18.1)	144 (16.0)	0.45	153 (18.1)	154 (16.8)	0.60
Moderate	206 (24.8)	237 (26.3)		227 (26.9)	237 (25.8)	
High	475 (57.1)	519 (57.7)		465 (55.0)	526 (57.4)	
**Healthy diet score[3]** (0-24)	12.7 (3.8)	12.0 (3.6)	**<0.001**	12.1 (3.8)	12.3 (3.8)	0.32
**Alcohol consumption[4]**						
Alc. dependency	8 (1.0)	3 (0.3)	0.23	6 (0.7)	2 (0.2)	0.26
Risk cons.	64 (2.9)	68 (7.6)		57 (6.9)	69 (7.5)	
Not at risk	742 (91.2)	829 (92.1)		759 (92.3)	846 (92.3)	
**Smoking**						
Daily	72 (8.5)	79 (8.8)	0.57	88 (10.2)	76 (8.3)	0.23
Occasionally	31 (3.7)	25 (2.8)		40 (4.6)	35 (3.8)	
Never/former	743 (87.8)	796 (88.4)		733 (85.1)	806 (87.9)	
**Waist (cm) m/f**						
>101/87	473 (56.2)	435 (48.8)	**<0.001**	476 (56.1)	469 (52.0)	**0.03**
94-101/80-87	184 (21.9)	269 (30.2)		190 (22.4)	253 (28.0)	
<94/80	184 (21.9)	187 (21.0)		182 (21.5)	180 (20.0)	
**Lifestyle index[5]**						
Group with available data as reference category	**Crude: OR 1.19 (95%CI 1.00-1.42), p=0.05**<br><br>Model 1: 1.19 (95% CI 1.00-1.42), p=0.05			Crude: OR 0.86 (95%CI 0,73-1.03), p=1.00<br><br>Model 1: 0.85 (95%CI 0.72-1.02), p=0.07		

Begränsad delning

**Alternative lifestyle index[6]**						
Group with available data as reference category.	Crude: OR 0.86 (95% CI 0.73-1.02), p=0.08<br><br>Model 1: OR 0.86 (95% CI 0.72-1.02), p=0.07			Crude: OR 0.90 (95% CI 0.76-1.07), p=0.24<br><br>Model 1: OR 0.87 (95% CI 0.73-1.03), p=0.11		

1817 individuals were included in the main analysis (have data on education and lifestyle index at baseline and 3-year follow up).
1715 individuals miss data on either of these variables (n=1350) or were lost to follow-up before the 3-year follow-up (n=365).
n (%) or mean (SD), Chi2-test for categorical variables, T-test for continuous variables and ordinal regression for lifestyle index.
Model 1: Adjusted for age, sex, and education.
[1] Basic: ≤9 years, compulsory level, mid-level: 10-12 schooling years, high: ≥13 years, university level
[2] Low: ≤60 minutes/week, moderate: 60-150 minutes/week, high: ≥150 minutes/week
[3]Calculated from questionnaire-data on four favourable food groups and four unfavourable food groups
[4]Based on the AUDIT questionnaire. Alcohol dependency: ≥16p (men), ≥14p (women), risk consumption: 8–15p (men), 6–13p (women), not at risk: ≤7p (men), ≤5p (women)
[5]Represents the sum of scores (1-3) on physical activity, alcohol, smoking and diet (with HDS categorized into tertiles)
[6]Represents the sum of scores (1-3) on physical activity, alcohol, smoking and waist
