# Supplementary material for: Effect of pictorial-based information about atherosclerosis on adherence to lifestyle recommendations: results from the VIPVIZA randomised controlled trial
Source: Open Heart. 2026 Jul 23;13(2):e004136. doi: 10.1136/openhrt-2026-004136 (PMC13404837; doi:10.1136/openhrt-2026-004136)
Supplement: online supplemental table 4 [file openhrt-13-2-s009.pdf]

Supplementary table 4. Missing data among participants in the 3-year follow-up.

Variable	Intervention<br>n=1580	Control<br>n=1587
Age	0	0
Sex	0	0
Education	14 (0.9)	17 (1.1)
Smoking at baseline	3 (0.2)	4 (0.3)
Smoking at 3 years	11 (0.7)	19 (1.2)
Physical activity at baseline	16 (1.0)	16 (1.0)
Physical activity at 3 years	74 (4.7)	67 (4.2)
Alcohol at baseline	34 (2.2)	38 (2.4)
Alcohol at 3 years	127 (8.0)	124 (7.8)
Diet at baseline	91 (5.8)	94 (5.9)
Diet at 3 years	506 (32)	478 (30.1)
Waist at baseline	16 (1.0)	30 (1.9)
Waist at 3 years	37 (2.3)	48 (3.0)

n (%)
